# An embarrassment of richnesses: the PFC isn’t the content NCC

**DOI:** 10.1093/nc/niae017

**Published:** 2024-05-18

**Authors:** Benjamin Kozuch

**Affiliations:** Philosophy Department, University of Alabama, Tuscaloosa, AL 35401, United States

**Keywords:** PFC, NCC, visual consciousness, richness, higher-order theory, working memory

## Abstract

Recent years have seen the rise of several theories saying that the prefrontal cortex (PFC) is a neural correlate of visual consciousness (NCC). Especially popular here are theories saying that the PFC is the ‘content NCC’ for vision, i.e. it contains those brain areas that are not only necessary for consciousness, but also determine ‘what’ it is that we visually experience (e.g. whether we experience green or red). This article points out how this “upper-deck” form of PFC theory is at odds with the character of visual experience: on the one hand, visual consciousness appears to contain copious amounts of content, with many properties (such as object, shape, or color) being simultaneously represented in many parts of the visual field. On the other hand, the functions that the PFC carries out (e.g. attention and working memory) are each dedicated to processing only a relatively small subset of available visual stimuli. In short, the PFC probably does not produce enough or the right kind of visual representations for it to supply all of the content found in visual experience, in which case the idea that the PFC is the content NCC for vision is probably false. This article also discusses data thought to undercut the idea that visual experience is informationally rich (inattentional blindness, etc.), along with theories of vision according to which “ensemble statistics” are used to represent features in the periphery of the visual field. I’ll argue that these lines of evidence fail to close the apparently vast gap between the amount of visual content represented in the visual experience and the amount represented in the PFC.

## Introduction

The human prefrontal cortex (PFC) is a crown jewel of natural selection. This furthermost anterior part of the brain rapidly expanded over the last two million years, enabling distinctive human abilities such as reasoning, long-term planning, and complex metacognition (Wills 1993, Fuster 2002). The PFC achieves this through the supervisory and coordinating role it plays in the brain, something that it is uniquely positioned to do because of its prolific and widespread connections to areas throughout the rest of the brain (Miller and Cohen 2001; Alvarez and Emory 2006).

More recently, the PFC has had yet another role attributed to it, this being that which makes conscious visual experience possible (e.g. Lau and Rosenthal 2011; Brown et al. 2019, Michel and Morales 2020). This ‘PFC Theory’ has been met with many early empirical successes, ones coming from numerous correlations found between visual experience and PFC activity (for review: Michel 2022). On the other hand, such evidence can be accounted for without hypothesizing the PFC to be essential for consciousness (Kozuch 2014; Pitts et al. 2014; but see Michel and Morales, 2020), and PFC Theorists have difficulty explaining why the PFC can be severely damaged without equally severe deficits in consciousness appearing (Pollen 1999, 2007; Kozuch 2014, 2021, 2023, Boly et al. 2017; but see Odegaard et al. 2017). Perhaps the debate over the PFC’s role in visual consciousness is not to be resolved soon. Nonetheless, I believe that the debate can be moved forward. More specifically, there is a strong case to be made for the idea that one of the two main forms of PFC Theory is false. I will explain.

Something interesting about the arguments that have been previously made against PFC activity being constitutive of consciousness (e.g. Lamme 2003, 2010; Block 2007, 2014) is that they usually don’t distinguish between a major division in types of PFC Theory, this being between “Upper-deck” and “Lower-deck” PFC Theory (Kozuch 2021; Raccah et al. 2021, Michel 2022). According to Upper-deck PFC Theory, the PFC actually ‘provides the content’ found in visual experience [it is a content neural correlate of consciousness (content NCC) (Chalmers 2000)], which means that visual properties are consciously experienced only when the PFC represents them; according to Lower-deck PFC Theory, the content that appears in consciousness is located in sensory parts of the brain, it is just that such content can’t become conscious unless it is properly connected with activity in the PFC.

This article is an argument against the more vulnerable of the two forms of PFC Theory, Upper-deck PFC Theory. Below, I raise two problems for it. The first comes from the fact that, if we look at the visual functions that the PFC carries out (e.g. working memory, metacognition, etc.), there appears to be a vast gulf between the number of representations that these functions produce and the amount we seem to find in visual experience. I’ll also argue that this continues to appear true even once we’ve taken into account data thought to challenge the apparent “richness” of visual experience (e.g. change and inattentional blindness). But if the PFC does not produce enough content to supply the entirety of our visual experience, then it cannot be the neural basis of the content of visual experience. The second problem for Upper-deck PFC Theory that I’ll point out comes from the fact that, even if one of these PFC functions represented enough visual properties to supply the content of visual experience, it is likely that the content being used in its operation is stored, not in the PFC, but rather in visual areas; in which case the PFC can’t be considered the content NCC for vision.

Of course, before these arguments could go through, the empirical evidence mentioned above would need to be addressed, i.e. the evidence sometimes taken to show that visual experience is not as rich as it seems (e.g. inattentional blindness, subjective inflation) (Simons and Chabris 1999; Mack 2003; Knotts et al. 2018). These are discussed in due course. However, even once these data are taken into account, it will look unlikely that visual experience is “sparse” enough for the PFC to be what supplies the content of visual experience.

The reader might notice similarities between the arguments given below and those given previously (e.g. Lamme 2003, 2004, 2010, Block 2007, 2014), since this article argues against PFC Theory by trying to show that the amount of content found in visual experience exceeds the amount of content that the PFC represents. However, the argument given here is novel and distinct in a number of ways, a partial list of these being: (i) The argument here is primarily against, not PFC Theory in general, but rather a subtype (‘Upper-deck’ PFC Theory), one that has the virtue of being its currently more popular form, but which has the vice of being more susceptible to disproof (as we’ll see below). (ii) The argument given here will not use some general, undefined notion of “richness” to argue against PFC theory, but rather will appeal to (and defend) some widely agreed upon claims concerning how visual experience appears in introspection. (iii) Finally, earlier arguments have so far only argued against the idea that the PFC functions of working memory and/or attention are constitutive of consciousness. But there is another PFC function that must be considered for this role: metacognition (Fleming et al. 2012). We’ll consider it below.

One might ask: Why does it matter if Upper-deck PFC Theory is false? First, while there do exist some forms of Lower-deck PFC Theory (e.g. Lau and Brown 2019, Fleming 2020), Lower-deck Theory seems at this time to be underdeveloped. If this is true, then if Upper-deck PFC Theory falters, the development of Lower-deck Theory will become that much more pressing. Second, PFC Theory is closely aligned with the philosophical theory of consciousness known as Higher-order Thought (HOT) Theory (Rosenthal 1997, Carruthers 2000). HOT Theory suffers from a well-known objection, the problem of the Mismatched HOT (Neander 1998, Block 2011; see Rosenthal 2011, Weisberg 2011), and many HOT Theorists have responded to this by adopting what we could call an upper-deck version of HOT Theory, a version whose fate (I argue below) is tied to that of Upper-Deck PFC Theory (Kozuch 2021); so, if Upper-deck PFC theory fails, this might require not just a new form of PFC Theory, but also of HOT Theory. Finally, the neuroscientific study of consciousness is still in its beginning stages, which means that there is the potential to squander time and resources chasing theories that are eventually shown to be false. This article shows why we should worry about this in the case of Upper-deck PFC Theory.

The article’s layout is as follows: The first section discusses background issues, including what the two main types of PFC Theory are, and what predictions they make. In the next two sections, we examine the issue of how much and what kinds of content there is in visual experience: In the first of these two, the issue is investigated using introspection, doing so by appealing to widely made claims concerning how visual experience appears when we reflect upon it; in the second of these two, we review evidence sometimes thought to show that visual experience contains less content than it appears to, arguing that this evidence doesn’t require us to give up any of the widely held claims about visual experience that were considered in the section just before. The final section of the article takes a close look at the kinds of function carried out by the PFC, finding that none of them are candidates for producing enough (or the right kind of) representations to supply our visual experience.

## Upper-deck PFC Theory and its predictions

As discussed above, this article aims to evaluate the Upper-deck form of PFC Theory. In this section, I clarify what PFC Theory is and describe its connection to the philosophical theory of consciousness known as Higher-order Thought Theory. Later in this section, I point out some conditions under which we should think that Upper-deck PFC Theory is false.

### Upper- vs Lower-deck PFC Theory

The idea that the PFC is essential for consciousness has grown in popularity in recent years (e.g. Dehaene et al. 2014; Kriegel 2007a; Lau and Rosenthal 2011; Lew and Lau 2017; Brown et al. 2019; Mashour et al. 2020; Michel and Morales 2020; Michel 2022). This ‘PFC Theory’ comes in two forms (Kozuch 2021; Raccah, Block and Fox 2021; Michel 2022). In its first form, Upper-deck PFC Theory, activity in the PFC actually ‘constitutes’ the content of visual experience. According to this version, when one experiences redness, it is because redness is currently being represented in the PFC (and if greenness had instead been represented, one would have experienced greenness, and so on). In this case, the PFC would be a NCC, or, more specifically, what David Chalmers has called a “content NCC” for vision, this being a neural system whose activity directly determines what content appears in visual experience (e.g. determines whether one experiences redness or greenness) (Chalmers 2000, Crick and Koch 2003; Hohwy and Bayne 2015, Kozuch and Kriegel 2015). This is often distinguished from the “background NCC” (Chalmers 2000); those neural systems that provide the overall state of consciousness, such as being awake, being in a dreamful sleep, and so on. In the second kind of PFC Theory, Lower-deck PFC Theory, when one has an experience of redness, the activity constituting one’s experience of redness will be located somewhere outside of the PFC (probably visual areas), but such content will be conscious only if it is connected with PFC activity in the right way, perhaps through neural synchrony (Varela et al. 2001; Melloni et al. 2023) or recurrent processing (Lamme and Roelfsema 2000). In the Lower-deck version, the PFC is not a content NCC, but rather is what could call an “enabling NCC,” a neural system such that its operating normally is necessary for our having any conscious content at all (cf. Chalmers 2000:18).

This article will only be concerned with the Upper-deck form of PFC Theory. This is currently the more popular form (Lau and Rosenthal 2011; Metcalfe and Schwartz 2016; LeDoux and Brown 2017, Brown et al. 2019, Dehaene et al. 2006; Mashour et al. 2020), though there are also adherents of the Lower-deck form (Lau 2008, 2019, Lau and Brown 2019, Cleeremans et al. 2020, Fleming 2020). Upper-deck PFC Theory also appears to be a commitment of a popular philosophical theory of consciousness, the Higher-order Thought Theory. I will explain.

According to HOT theory, a mental state becomes conscious when and only when it is represented by another belief-like mental state (e.g. Gennaro 1996, Rosenthal 1997, Carruthers 2000). Because of the kind of cognitive complexity such states involve, the PFC is the most likely place to produce HOTs (Lau and Rosenthal 2011, Lau 2011; see esp. Kozuch 2023; but see Gennaro 2012), and if this is true, then PFC Theory and HOT Theory would be allied. But consider now a well-known objection to HOT theory, one that we can call the “Problem of the Misrepresenting HOT” (Neander 1998; Block 2011). The objection concerns instances where the content of the higher-order (HO) and lower-order (LO) states conflict, e.g. a case where the LO state represents greenness, but the HO state represents the LO state as representing redness. There is a vast literature concerning how HOT theory should handle the dilemma that this creates (Carruthers and Gennaro 2020), but this need not detain us: all that is important here is that the objection forces HOT theorists to choose which color the subject will consciously experience: will it be the greenness of the LO state, or the redness of the HO state? (That is, will the subject have the kind of phenomenal experience that one typically has when looking at green-surfaced objects or red-surfaced objects?) To my knowledge, all HOT Theorists (save Wilberg 2010) have so far chosen to hold views according to which the experience of color corresponds to the HO state (Rosenthal 2011, Weisberg 2011, Brown 2012, 2015; Berger and Brown 2021); that is, they adopt an “Upper-deck” form of HOT theory (cf. Block 2023, Ch. 12), this being in contrast to the Lower-deck form, the one according to which the subject has a color experience corresponding to the content of the LO state. Notice now that, since the subject’s experience corresponds to the content of the HOT, and since the PFC is widely held to be the most likely place to produce HOTs, it looks as if Upper-deck HOT theory ‘entails’ Upper-deck PFC Theory; if so, we can consider Upper-deck HOT Theory and Upper-deck PFC Theory to share fate.

As said above, we are only concerned with Upper-deck PFC Theory, and all mentions of “PFC Theory” below refer to the Upper-deck version, unless noted. Now we move on to discuss what predictions PFC Theory has to make.

### PFC Theory and the content of visual experience

According to (Upper-deck) PFC Theory, all content found in visual experience is conscious in virtue of its having been represented in the PFC (since the PFC is the content NCC for vision). If this is true, then PFC Theory predicts that, in each moment, the PFC must represent all of the same content as is found in visual experience (in addition to whatever other content it might represent). Here’s the idea:

Parity Thesis: At any given moment, for each representation with content C appearing in visual experience, there is a representation with content C instantiated in the PFC.

Since PFC Theory entails the Parity thesis, this gives us a way to gauge the plausibility of PFC Theory: we should take PFC Theory to be likely to be true only if we think the same of Parity.

What comes below is an argument against Parity. Making this argument requires knowing two things: (i) how much and what kinds of content appear in the typical visual experience, and (ii) how much and what kinds of visual content the PFC routinely represents. The next two sections are dedicated to investigating the former issue, and the section after that to the latter.

## How visual experience appears in introspection

Our investigation of the issue of what content appears in the typical visual experience begins with the following question: if we introspect on visual experience, what kinds of content does it ‘appear’ to contain? However, before looking at this question, I should discuss what kind of evidential role we should take introspection to play in NCC research. I start by giving some context.

Soon, we begin the task of determining how visual experience appears in introspection. We do this by building what I’ll call the “Introspective Picture” of visual experience, this being an accounting of how much and what kinds of content visual experience appears to have, when introspected upon. The Introspective Picture is constructed out of what we can call “introspective judgments,” these being introspectively formed beliefs concerning what content is in one’s visual experience. While it is true that there have been arguments made against the reliability of introspection in general (e.g. , esp. Ch. 11; Cohen et al. 2016), these arguments appear to have been addressed adequately elsewhere (Haun et al. 2017). Whether this is true or not, this debate can be set aside, since all that is needed for the argument that I give below is that at least ‘some’ introspective judgments have prima facie plausibility (see also Bayne and Spener 2010). Now I explain why we should think that they do.

The explanation starts with us considering how introspective judgments can ‘count as evidence’ in NCC research. This is the thesis that I have in mind:

An introspective judgment that one’s visual experience has feature F (e.g. it is informationally rich) has the potential to count as evidence for their visual experience actually having F.

This relatively weak thesis must be true: if we didn’t take ‘some’ introspective judgments to have ‘some’ chance of being true, we couldn’t set the explanandum for a science of visual consciousness. For example, the only reason that one of the goals of NCC research is to explain color consciousness is because people make introspective judgments saying that they consciously experience colors; conversely, since no one ever reports experiencing electromagnetic radiation, it is not a goal of NCC research to explain experiences of electromagnetism. The upshot: each introspective report, i.e. each report saying that visual experience has feature F, has at least the potential to have evidential value for NCC research; more specifically, it can count as evidence for visual experience actually having feature F.

The question now is: which introspective judgments should we take to have this kind of evidential force? The issue is not simple here, since researchers often disagree as to what the deliverances of introspection are, with the ensuing debates often being difficult to resolve (Bayne and Spener 2010, Kriegel 2015, Ch. 1). Here, we tread the path of minimal controversy, doing so by appealing just to those introspective judgments upon which many researchers independently converge, and which have been largely or completely non-controversial. We’ll refer to these as “well-justified” introspective judgments [cf. Bayne & Spener’s “trustworthy judgments” (Bayne and Spener 2010)]. I propose now that, because well-justified introspective judgments have been converged upon in this way, they can play the role in NCC research that I described just above; that is, I’m proposing the following thesis:

If a well-justified introspective judgment states that visual experience has some feature F, then this counts as evidence for visual experience actually having F.

This thesis seems plausible, given that we know (for reasons stated above) that there must be at least some introspective judgments that have evidential value in NCC research. For fulfilling this role, we have no better candidate than well-justified judgments, i.e. those introspective judgments that consciousness researchers have converged upon, and about which there’s been little or no controversy. Of course, introspective judgments—even when well-justified—are ‘defeasible’ and therefore might be shown to be false by contrary and outweighing arguments or data; this is why a later section of the article will look at the issue of whether there is any such evidence. Nonetheless, the point still stands: we can consider a well-justified introspective judgment stating that visual experience has feature F to be evidence for visual experience having F. I think that we can go further than this, however, since it is intuitive to think that an introspective judgment that visual experience has F gives reason to think that it ‘actually has’ F, until the appearance of contradictory evidence.

Now I’ll present three well-justified introspective judgments concerning visual experience. We look at each in turn.

### Visual experience contains numerous representations

One idea upon which many consciousness researchers have converged is that, if one introspects upon visual experience, it appears informationally rich, in that it seems to contain a great deal of content: it appears to include numerous representations of visual properties (e.g. colors, shapes), with these properties being represented in numerous and varied parts of the visual field (Siewert 1998, Ch. 7; Gregory 1966, Searle 1992, Vogel and Machizawa 2004, Chuard 2007, Tononi et al. 2016, Haun et al. 2017, Knotts et al. 2018, Kozuch 2021, 2023). Even researchers who argue against visual experience actually being rich in this way agree that it at least ‘appears’ rich (Cohen et al. 2016; Blackmore et al. 1995, O’Regan 1992; but see Jaynes 2000). Additionally, subjects carrying out tasks related to visual iconic memory indicate that their visual experience appears detailed and rich (Sperling 1960; Baars 1988, Tye 2006, Block 2007), and there are experimental results suggesting that the average person takes visual experience to make large amounts of information available to them (Levin et al. 2000).

Given all these, it seems that a well-justified judgment (in the above sense) concerning how visual experience appears in introspection would be one saying that it is informationally rich. This raises the question as to precisely what kinds of content we should take there to be within the typical visual experience. Here, the only kind of visual property with which we’ll be directly concerned is ‘sensory’ properties. This includes those lower-level properties that researchers have frequently posited to be represented within visual experience (Bayne and McClelland 2019), the ones most often cited here being color, shape, and spatial relations (Brogaard 2013; Prinz 2012, Ch. 5; Peacocke, 1983, Dretske 1995, Tye 1995, Lormand 1996), with some researchers adding the properties of brightness, texture, and motion (Siegel and Byrne 2017; Bayne and McClelland 2019). Sensory properties can be distinguished from what we’ll call “perceptual” content, representations of higher-level visual properties such as someone appearing to be in a certain emotional state, or one event having caused another (Siegel and Byrne 2017). Among the perceptual properties that will be of particular importance below is what are called “categorial” properties (Kriegel 2007b; Siewert 1998, Ch. 7), perceptual representations representing something as being of a certain type (or as having a certain identity). An example here would be someone visually representing something as being not just yellow and crescent-shaped, but also ‘as being a banana.’ Given the controversy surrounding whether perceptual properties are actually consciously experienced—that is, whether they are ‘phenomenally’ experienced (Siegel and Byrne 2017)—we will not be appealing to perceptual properties in the argument against PFC Theory given below.

As seen above, researchers take there to be a wide variety of sensory properties that are represented in visual experience. Here, we mostly focus on just a subset, this being color, shape, and motion. In the case of color, it must in turn be understood as consisting of three components, those of brightness, hue, and saturation (Tkalčič and Tasic 2003; Lotto and Purves 2000; Long et al. 2006). We focus only on brightness and hue below.

Observe now that it is not that visual experience simply represents sensory properties such as shape and color as being out there ‘somewhere’ in the environment, but rather that visual experience represents them as appearing ‘at some location’ in the visual field (Clark 2000, Ch. 5, 2004). For example, colored surfaces are not represented simply as being out there in the environment, but rather as being some specific distance and/or direction from the subject, e.g. a red surface might be represented as being at eye-level, directly in front of the subject, and at a distance equal to a meter.

We just cataloged the kinds of properties that appear to be represented in visual experience; now we turn to the issue of how ‘frequently’ each of these properties is represented in the typical visual experience; that is, how many instantiations of each type of sensory representation the typical visual experience contains. As seen above, the commonly held view is that visual experience contains abundant sensory representations (e.g. Carruthers 2000, Ch. 9, Wolfe 1999, Haun et al. 2017; Cohen et al 2016, Knotts et al. 2018, Kozuch 2021). Put more precisely, according to this conception of visual experience, the typical visual experience contains many instantiations of representations of sensory properties (hue, brightness, shape, etc.), properties that are each represented as occurring in some location in the visual field. Consider, as an example, a case in which one sees a banana along with other fruit located on a table in a sparsely furnished art gallery: here, one’s visual experience might contain representations of the shape and color (i.e. brightness and hue) of the banana in one part of the visual field; this is along with the shape and color of other fruit located on the table in another part of the visual field; all these are accompanied by a representation of the shape/color of the table upon which the fruit sits, perhaps along with representations of the shape/color of the art that is located on the wall behind the table; and so on.

At this point, we can introduce a very general thesis concerning visual experience, one that captures an important aspect of the Introspective Picture of visual experience, and which enjoys wide support in the literature:

Numerosity: Visual experience contains the representation of a substantial number of sensory properties (hue, brightness, shape, etc.), ones that are represented in many different parts of the visual field.

Now we move on to look at two other theses deserving inclusion within the Introspective Picture.

### Visual experience contains finely grained representations of what appears in central vision

Visual experience seems to include an especially detailed representation of what appears in central vision, one that represents many of the properties appearing there, and which uses especially fine-grained representations to do so (Carruthers 2000, Ch. 11, Tye 2006, Chuard 2007, Siewert 1998, Ch. 7, Kozuch 2021). Support for this idea can be found by thinking about how, in cases where you focus your eyes on an object—say, the palm of your hand—your visual experience appears to contain a tremendous amount of detail within central vision, in that you seem to be “aware, simultaneously, of a network of fine lines and wrinkles, and of subtle texture and colour gradients” (Carruthers 2000, Ch. 11:300).

The point here would be that central vision, on its own, seems to contain numerous sensory representations. Additionally, and perhaps more importantly, the representations of what appears in central vision are exceptionally ‘fine-grained’: things such edges, surfaces, and textures appear represented with a high degree of detail; this, in turn, implies that representations in central vision are especially informationally rich.

This widely held thesis merits inclusion in the Introspective Picture of visual experience; we can understand it as follows:

Central Vision: Visual experience includes numerous, fine-grained representations of what is located in the center of one’s visual field.

Given its wide support, Central Vision seems to be another well-justified introspective judgment. Now we consider one more.

### Visual experience includes a representation of color and hue throughout the visual field

If one introspects on the typical visual experience, one finds that, for each part of the visual field, there is a representation of the color (hue and/or brightness) of whatever surface is visible there (Chuard 2007, Tononi et al. 2016, Haun et al. 2017, Knotts et al. 2018, Kozuch 2021, 2023). For example, if we examine the visual experience that one has when standing on the beach, facing the ocean, it might look as follows: throughout one large, lower part of the visual field, the color of the sand is represented, interrupted only by the colors of the surfaces of whatever objects are located there; a little higher in the visual field, one finds the color of the water, its translucent green punctuated by the white of the foam of its breaking waves; in another part one finds the blue of the sky, interrupted by whiteness where clouds are located; and so on.

These thoughts suggest our third and final thesis:

Expansive Color: Visual experience includes a representation of brightness and/or hue in each part of the visual field.

We’ve now seen three theses to be included in the Introspective Picture, each of which seem to be a “well-justified” introspective judgment, i.e. a judgment such that, because it is an uncontroversial thesis concerning what visual experience looks like in introspection, it is evident for visual experience having whatever feature the introspective judgment attributes to visual experience. Now we move on to consider evidence thought to undercut the Introspective Picture.

## Evidence against the richness of experience

There are many data and arguments thought to undermine the idea that visual experience is informationally rich. These include experimental phenomena such as change and inattentional blindness (Rensink et al. 1997, Simons and Chabris 1999, Mack 2003) and inattentional inflation (Knotts et al. 2018), along with the theory stating that our visual experience contains generic phenomenology (Kouider et al. 2010). Let’s collectively refer to these data and theories as the “anti-richness data.” If the anti-richness data are able to show that visual experience contains less content than it seems to in introspection, this helps PFC Theory, since this means that the PFC wouldn’t need to produce as much content for it to be considered a candidate for supplying our visual experience. In this section, I argue that the anti-richness data do not require thinking that the typical visual experience contains any less content that it is said to have in the Introspective Picture.

The debates concerning the richness of visual experience are contentious, and the literature deep, and so the goal cannot be to come to ‘definitive’ conclusions concerning how much content visual experience typically contains. Rather, the intention will be just to show that the anti-richness data are consistent with the Introspective Picture: since the introspective judgments that compose the Introspective Picture are well-justified and therefore count as evidence for visual experience actually being like how it is described in the Introspective Picture (see the section entitled “How visual experience appears in introspection”), this provides prima facie grounds for preferring explanations of the anti-richness data that preserve the truth of the Introspective Picture; this, in turn, will give us at least tentative reason to think that something like the Introspective Picture is true. This is good enough to motivate moving on to the main issue of the article, that of whether the PFC might supply the content of visual experience.

Now we turn to analyzing the anti-richness data. The various types of evidence are grouped according to how the advocate of the Introspective Picture could respond to them.

### Differences but not absences of content (inattentional inflation)

According to the theory of inattentional inflation, a lack of attention to a peripherally presented stimulus makes subjects overly confident (more prone to false positives) when making judgments about the stimulus (Knotts et al. 2018). In a representative experiment (Rahnev et al. 2011), subjects indicated whether a grating was presented in a location that was either attended or unattended. Subjects not attending to the location were more prone to false positives than subjects attending to the location (see also Solovey et al. 2015, Li et al. 2018). These results have been claimed to conflict with the idea that visual experience is rich (Knotts et al. 2018).

Inattentional inflation, however, does not provide reason to think that visual experience contains any less content than it is held to in the Introspective Picture. This is because, in the way that these experiments are often interpreted (Knotts et al. 2018; but see Odegaard et al. 2018), it is not hypothesized that there is any ‘absence’ of content from the subject’s visual experience, just a ‘difference’ in content. Consider that, of those subjects erroneously indicating that a grating appeared in the periphery, the advocates of inattentional inflation have usually not claimed that those subjects experience ‘nothing’ in that part of the visual field, but rather that they illusorily experience a grating to be there (instead of the uniform background color that actually appears there). Indeed, advocates of inattentional inflation are often careful to point out that their claim is not that visual experience contains any less content than it appears to, but rather just that the typical visual experience involves more “filling in” of details than we might have thought, with what is experienced in the periphery often being determined by expectations (Knotts et al. 2018). If so, inattentional inflation does not provide reason to think that visual experience contains any less content than it seems to in introspection and therefore is not of help to PFC Theory. Additionally, this not the only plausible explanation of inattentional inflation that doesn’t require us to hypothesize any lack of visual content. According to another alternative, the subject experiences, in the part of the visual field where the grating failed to appear, not a filled-in grating, but rather what actually appeared there, the uniform color of the background; the subject is just mistaken in their belief that they experienced a grating.

On the other hand, it is true that the two explanations given so far are not the only ones available. Some researchers (Odegaard et al. 2018) have argued that inattentional inflation might manifest as a tendency for peripheral representations to be “subjectively misestimated to be rich in content” (p. 2), with this apparently meaning that peripheral experience is not as detailed as it seems. But such a possibility—one in which the subject seems to have a scotoma-like absence of visual experience where the stimulus failed to be presented (cf. Kozuch 2019; Wu 2014, Ch. 4).—certainly seems no more likely than the other two explanations already discussed.

Putting aside the question of which explanation is correct here, it seems that we at least have reason to think that inattentional inflation is consistent with the Introspective Picture, which is enough for present purposes.

### Absences of perceptual content (inattentional and change blindness, generic experience)

Remember the distinction made above between sensory and perceptual representations (see the section entitled “Visual experience contains numerous representations”): sensory representations are those associated with lower-level kinds of visual processing (e.g. color, shape, motion), whereas perceptual representations are associated with higher-levels of visual processing (e.g. object identity, causal relations). This distinction is important to keep in mind when considering anti-richness data, since some of them are plausibly construed as being absences of just perceptual, and not sensory, content.

This includes those experimental phenomena that have been most widely appealed to in anti-richness arguments, these being change and inattentional blindness (Rensink et al. 1997, Simons and Chabris 1999, Mack 2003). These experiments present examples of subjects failing to notice seemingly conspicuous events in their visual field, such as a person in a gorilla suit beating their chest or a centrally located jet engine continuously disappearing and reappearing. However, while numerous researchers appeal to these studies to argue that visual experience is relatively impoverished (O’Regan 1992, Rensink et al. 1997, Simons and Levin 1997; Simons 2000; Weisberg 1999; Nöe & O’Regan 2000; O’regan & Noë 2001, Mack 2003, Gennaro 2004, Dehaene et al. 2006), these experimental phenomena are most plausibly interpreted as involving only an absence of perceptual, and not sensory, content. For example, these results don’t give any reason to think that the inattentionally blind subject lacks an experience of shape, color, motion, etc. throughout the visual field (Wolfe 1999; cf. Block 2007), this being true even in the case of those properties possessed by the object to which the subject is inattentionally blind (e.g. the gorilla’s shape, color, and motion) (Knotts et al. 2018; cf. Wu 2014, Ch. 4). Rather, the only anti-richness conclusion that these data mandate is that there is a lack of a ‘perceptual’ representation of the gorilla, more specifically, that there is a lack of a ‘categorial’ representation, one that represents the consciously perceived black moving form ‘as’ a gorilla. The same sort of observation can be made in the case of change blindness, in that there is no lack of sensory properties within the subject’s visual experience; instead, it is just that the subjects fail to perceptually represent the change that is occurring in the target object (e.g. the jet engine appearing and disappearing) (De Brigard and Prinz 2010; see Knotts et al. 2018).

Another way that researchers have argued against richness is by appealing to the possibility of “generic experience” (Kouider et al. 2010). This idea is largely inspired by a study by De Gardelle et al. (2009) in which it was shown that subjects in a partial report paradigm (Sperling 1960) would not notice if one of the letters in an uncued row were rotated (see also Kouider and Dupoux 2004). On one interpretation (De Gardelle et al. 2009), subjects do not experience each of the individual letters; instead, the subjects’ expectation that there would be an upright letter means that they experience the letter as being upright (p. 572). But just like in the case of inattentional inflation, this would not entail there being any absence of content in their experience, just a difference: instead of experiencing a rotated letter, they experience one that is right-side-up. However, according to later interpretations of these studies (Kouider et al. 2010; cf. Byrne et al. 2007, Phillips 2016), subjects have a ‘generic’ experience of the letters: the characters in the display are experienced by the subject, but in way where the subject does not experience the character as having any particular identity (e.g. they do not experience it as being the letter “A”). But this explanation only hypothesizes there to be a lack of perceptual, and not sensory, representations; more precisely, a lack of a ‘categorial’ representation. According to this way of understanding the idea, if a subject is having a generic experience of the letter “A,” it seems that the subject would be experiencing many of the A’s sensory properties (e.g. its shape, position, color, etc.) and would only fail to experience one of its perceptual properties, in that they would fail to experience it ‘as’ an “A.” Again, there is no need to posit any less sensory properties.

One other line of anti-richness evidence should be discussed, a form of inattentional blindness not fitting neatly into the two categories discussed above. This comes from a study by Cohen et al. (2020) in which subjects were given a short time (20 s) to explore a virtual environment (e.g. a construction site). On some trials, the experimenters gradually desaturated the peripheral visual field (i.e. turned it from color to black-and-white). Remarkably, some of the subjects would fail to notice this, and the experimenters take these results to “demonstrate a surprising lack of awareness of peripheral color in everyday life” (p. 13 842).

Certainly, such results are testament to how seemingly conspicuous changes in the visual field can go unnoticed if they occur outside of attention, at least in real-world type conditions in which “spatial attention is thought to ‘*tightly*’ track the current and upcoming fixation location” (p. 13 824). But, like in the cases of inattentional and change blindness examined above, it is not mandatory to hypothesize there to be any lack of sensory properties in the subject’s experience. Instead, it could be the case that subjects fail to perceptually represent that the arrangement of colors experienced in peripheral vision has changed from consisting of both hue and brightness to just brightness (cf. Knotts et al. 2018), but their experience is otherwise mostly normal, in the sense that it continues to satisfy the Introspective Picture; that is, the subjects continue to experience things such as numerous properties throughout the visual field (the thesis of Numerosity) and a comprehensive representation of color throughout the visual field (Expansive Color).

One might object here, however, saying that we can take the fact that such subjects fail to notice the desaturation of the peripheral visual field to mean that they were not experiencing hue and brightness there. But such an objection is based upon the idea that there is an entailment from a subject S not noticing X to S not experiencing X. While history shows that some researchers find this kind of entailment plausible (e.g. Simons and Chabris 1999), it begs the question against one holding that such subjects have a phenomenal experience (in the Blockian sense) of sensory properties throughout the visual field but are prevented from noticing that their color experience has changed because of a failure to access the peripheral color representations (Block 1995). This latter interpretation gains plausibility if one considers the fact that, if the subject’s attentional focus is so narrow as to prevent them from noticing the desaturation of the periphery, it might very well also be so narrow as to prevent them from noticing that their experience of hue and brightness throughout the peripheral visual field has become one of just brightness.

All in all, it seems that the Cohen et al.’s study can be taken to be at least consistent with the Introspective Picture, which is all that we needed to accomplish here. As discussed above, this is enough to make it worthwhile to ask whether the PFC might supply all of the content that appears in visual experience.

In this section, we’ve reviewed experiments and data thought to show that visual experience contains less information than it seems to in introspection, something that might prove helpful to PFC Theory. As just shown, each of the anti-richness data can be interpreted in ways consistent with the Introspective Picture, and—as discussed in this section’s introduction—this is enough to provide tentative reason to think that something like the Introspective Picture is correct. This means, of course, that it remains a possibility that the PFC Theorist might produce arguments showing that, though the anti-richness data is consistent with the Introspective Picture, the best explanation of these data includes the idea that the Introspective Picture is false in one or more ways. We return to this topic in the article’s conclusion. Now we move on to the main issue of the article, that of whether the PFC might supply the content of experience.

## Is the PFC the content NCC?

What we have found so far is that there is at least tentative reason for thinking that something like the Introspective Picture is correct, and that this remains true even once we have considered the anti-richness data. In this section, we assume that the Introspective Picture is mostly correct, the goal now being to see whether this implies that PFC Theory is false.

Remember the Parity Thesis, which says that, if the PFC is the visual content NCC, then, at any given moment, the PFC must represent all of the same content as is currently appearing in visual experience. Now we combine Parity with the three claims of the Introspective Picture, creating three predictions of PFC theory, saying how much and what kinds of content the PFC must routinely represent, if PFC Theory is true. The three claims:

Numerosity: In any given moment, the PFC represents a substantial number of sensory properties (hue, brightness, shape, etc.), ones that are represented as being in many different parts of the visual field.

Central Vision: In any given moment, the PFC instantiates numerous, fine-grained representations of what is located in the center of the visual field.

Expansive color: In any given moment, the PFC represents there to be some color and/or brightness in each part of the visual field.

It will be helpful to have a way to refer to these theses collectively, so we’ll call them the “PFC Predictions.”

It is my hope that the PFC Predictions prove theoretically useful beyond just this article, providing a general test for PFC Theory. The idea here is that, if PFC Theory is to be considered plausible, then we should have reason to think either that the PFC Predictions are fulfilled, or that visual experience is substantially less rich than it is in the Introspective Picture. In this section, I show why the first disjunct is false, thereby leaving PFC Theory with just the second option.

The argument proceeds by looking at the kinds of function that the PFC carries out, and then asking whether any are good candidates for supplying the content found in visual experience. What we try to determine, more specifically, is whether there is a “Sufficient Function”:

Sufficient Function: a function such that, because of the nature of its processing, it might produce enough and the right kind of visual representations to satisfy the PFC Predictions.

Because of the Parity Thesis, which says that the PFC represents all of the content found in visual experience, PFC Theory is true only if the PFC has a Sufficient Function. This gives us one way to show that PFC Theory is false, which is by showing that there is no Sufficient Function.

However, since what we are concerned with here is ‘Upper-deck’ PFC Theory specifically, there is another way to show that PFC Theory is false. This shows that, even if there is a Sufficient Function, it operates in a Lower-deck fashion; i.e. though the function in question produces all the content necessary to satisfy the PFC Predictions, the representations that appear in consciousness are not located in the PFC, but rather just manipulated by it. And so, it would not help PFC Theory if the capacity of, e.g. working memory was large enough to supply our visual experience, but the representations that it used were all in visual areas, rather than in the PFC.

Below, I present reason to think that PFC Theory is false in both of the above ways: in the next two sections, we’ll see why those PFC functions that are candidates for being a Sufficient Function probably don’t produce enough content to satisfy the PFC Predictions, and in the section after those, two we’ll see why each of these functions probably also operates in a Lower-deck fashion. First, we examine the issue of what PFC functions are candidates for being a Sufficient Function.

### PFC functions that are candidates for producing visual experience

Characterized at a general level, the function of the PFC is thought to be “cognitive control,” a kind of top-down executive processing that works to promote flexible, goal-directed behavior, usually at the expense of more habitual responses (Miller and Cohen 2001, Friedman and Robbins 2022). The PFC does this by carrying out a suite of higher-order cognitive abilities, ones traditionally thought to fall under three general categories: working memory, cognitive flexibility, and inhibition (Miyake et al. 2000; Lehto et al. 2003, Diamond 2013). The list of functions that the PFC is thought to carry out has more recently grown to include others, such as top-down attention, action-monitoring, metacognition, and multi-tasking (Miyake et al. 2000, Friedman and Robbins 2022).

The question now is, of the numerous functions that the PFC carries out, are any of them candidates for producing visual experience; that is, are any of them a Sufficient Function? Many PFC functions can be ruled out quickly, on the grounds that they do not involve producing visual representations at all; for example, the functions of inhibition and action-monitoring involve representing not visual properties, but rather upcoming or ongoing actions. There are, however, three PFC functions that produce significant amounts of visual representations, these being working memory, top-down attention, and metacognition. Next, we consider each. What we will find is that it is both the case that these functions probably don’t produce enough content to meet the PFC Predictions, and that each of these functions probably operates in a lower-deck fashion.

### Top-down visual attention and visual working memory

Top-down visual attention is the ability to prioritize the processing of certain stimuli out of the large amount of visual information with which we are presented at any given moment (Carrasco 2011, Moore and Zirnsak 2017). Top-down visual attention (VA) is what allows us to pick out a behaviorally relevant object out of a crowded and perhaps chaotic visual field (e.g. when finding someone at a concert). Visual working memory (VWM) is the ability to maintain visual information so that it can be used in service of ongoing tasks (Baddeley 2003, Luck and Vogel 2013). VWM allows us to maintain an object’s identity when eye movements cause it to appear in a different part of the visual field (Irwin 1992, 1996), or to notice when an object in the visual field has changed (e.g. in its orientation). VA and working memory both produce visual representations and therefore are candidates for being a Sufficient Function. However, each of these functions does not seem to produce enough or the right kind of representations to satisfy the PFC Predictions. Let’s see why.

In the case of both top-down VA and VWM, there are two general forms, one more focused on processing individual objects, and the other on more global kinds of information from the visual field (Treisman 2006; Brady and Alvarez 2011). We look at each in turn.

In the case of the object-based forms of VA and VWM, their capacity is far too low to satisfy the PFC Predictions (cf. Block 2007, 2014): the number of individual locations or objects able to be tracked in VA is typically limited to four or five, though it can reach eight if stimuli are widely spaced and presented alone (Alvarez and Franconeri 2007, Scimeca and Franconeri 2015). Estimates of VWM capacity hover around three or four items (Luck and Vogel 1997, 2013; Vogel and Awh 2008, Cowan 2010, Chun 2011), examples of an “item” being an object’s property such as its shape (Alvarez and Cavanagh 2004) or color (Luck and Vogel 1997, Vogel and Machizawa 2004). Under certain circumstances, the item-capacity of VWM can—in effect—be made larger, such as when training allows multiple properties to be chunked so that they take up just one of the three to four “slots” of VWM (Luck and Vogel 1997). An example here would be our ability to store two colors together if they frequently co-occur (Brady and Konkle 2009). A capacity of 24 is even sometimes achieved if the stimuli have some kind of identifiable higher-order structure to them (e.g. a set of same-colored circles arranged in a row) (Brady and Tenenbaum 2013). It is, of course, one thing to demonstrate such a high capacity in an experimental setting—one in which the visual stimulus is artificially sparse and well-organized—and another thing to provide reason to think that it would obtain with real-world visual scenes. In any case, even a capacity of 24 falls far short of the number of representations there seems to be in the typical visual experience, i.e. far short of the numerous experiences of color, texture, size, motion, etc. that the theses of Numerosity and Central Vision hypothesize the typical visual experience to contain.

We now move on to the second, more global form of VA and VWM, the one in which properties in the visual field are represented more schematically. The global form of VA is what is known as “distributed” attention (Srinivasan et al. 2009), which allows the visual scene to be processed in a more general, holistic manner (Oliva and Torralba 2006). Distributed attention makes possible the extraction of a scene’s “gist,” this being its more general characteristics, such as what the environment is (e.g. whether it’s a beach or street scene) (Oliva and Torralba 2001), or whether certain types of objects are present in the scene (e.g. animals or faces) (Evans and Treisman 2005, Evans and Chong 2012). Importantly, distributed attention provides the information to VWM that allows it to build what are known as “ensemble representations” (Alvarez 2011). These are representations produced by taking a number of individual measurements of some property in the visual field (e.g. the brightness of visible surfaces), then collapsing all of the measurements to one value (a “summary statistic”), often their average (Chong and Treisman 2003, Alvarez 2011). The visual system creates ensemble representations of many visual properties, such as orientation, location, gaze, and direction of motion (Bayne and McClelland 2019). Ensemble representations are cognitively advantageous since they exploit redundancies in the environment to produce compressed representations of visual information in the visual field, in effect accounting for a lot of the information from the visual field without taking on the computational demands of storing it all (Alvarez 2011).

It has been recently argued that ensemble representations show how VA/WM might provide all of the content found in our apparently rich visual experience, the idea being that much of what appears in the visual field is represented using summary statistics (Cohen et al. 2016). According to this idea, which we’ll call ‘Ensemble Theory’, only those items at the center of attention and the visual field are perceived with high resolution (and are held in the object-based forms of VA or VWM), while other parts of the visual field are represented using summary statistics. Perhaps Ensemble Theory has the potential to help show how VWM/VA could fulfill the PFC Predictions. For example, one might hypothesize that ensemble representations provide the mechanism by which there could be a comprehensive representation of hue and/or brightness throughout the visual field, thereby fulfilling the thesis of Expansive Color.

There are, however, reasons to think that Ensemble Theory is not able to account for visual experience. I will explain. According to Ensemble Theory, if one experiences a collection of e.g. peripherally located circles, this experience is supposed to be constituted by an ensemble representation. But now let us now consider a case in which the circles are of disparate size, asking the following question: what size will the subject perceive each individual circle to have? Here, it seems that Ensemble Theory would have to predict that each circle will be experienced as having the “same” size, i.e. whatever size is equal to the average size of the collection of circles. To see why, consider a foundational principle in NCC research, the “Isomorphism Constraint,” which says that, if some representation R is the neural basis of experience E, then R and E must match in their content (Noë and Thompson 2004). The Isomorphism Constraint is pretty innocent: it just rules out certain metaphysical oddities from arising, such as cases where a subject’s experience of a straight line is constituted by a representation of something other than a straight line, say, a representation of a crooked line (Kozuch and Kriegel 2015).

Getting back to our example, the Isomorphism Constraint means that what size the subject consciously experiences each circle to have must be determined by whatever representation underlies their experience of the circle. Since this is hypothesized to be the ensemble representation, and because the content of the ensemble representation is just one value (the circles’ average size), this seems to entail that the subject experiences each circle as having the same size (the average). This is inconsistent with the phenomenology: when looking at a collection of disparately sized circles, we seem to perceive them as having different sizes, with them seeming to be of roughly the same relative sizes that they actually have. And so it seems that Ensemble Theory has difficulty accounting for our experience of disparately sized objects that are peripherally located. Similar arguments can be made in the case of the other sensory properties that are said to be represented by summary statistics, such as orientation, location, motion, and others.

So far, we’ve seen reason to think that Ensemble Theory might be false. We also lack strong reason for thinking Ensemble Theory is true, insofar as the evidence currently available falls far short of suggesting that the visual system produces enough ensemble representations to fully populate our visual experience: if we survey the evidence concerning Ensemble Theory that is relevant to present purposes, what we find is that it so far only demonstrates subjects to be able to form ‘one’ ensemble representation at a time about ‘one’ specific (sensory) property, where this property is task-relevant and therefore subject to focused attentional resources (e.g. Dakin and Watt 1997; Ariely 2001, Parkes et al. 2001, Chong and Treisman 2003, Bauer 2009). Such evidence certainly shows that Ensemble Theory (as a theory meant to explain peripheral conscious vision) has some chance of being true, since it shows that subjects do in fact form ensemble representations. (If we couldn’t find any evidence that subjects produce ensemble representations, Ensemble Theory would be dead-on-arrival.) At the same time, this evidence is far short of showing that we should think that Ensemble Theory is true: for this, we’d need something further, i.e. evidence giving us reason to think that subjects routinely form enough and the right type of ensemble representations to supply something like what our peripheral visual experience appears like; we’d want evidence suggesting that the visual system is, at any given time, forming multiple ensemble representations of a number of distinct properties in a number of distinct portions of the visual field; furthermore, we’d want evidence suggesting that these representations are formed about properties in the visual field that are not currently subject to focused attentional resources (in contrast to how they are in the experiment). It seems, then, that the evidence for Ensemble Theory is properly interpreted as merely opening the door to the theory potentially being shown true in the future, but not as giving reason to think that it is likely to be true.

Overall, the functions of VWM and VA, both in their object-based and more global forms, look unlikely to produce enough or the right kind of representations to be what supplies our visual experience (i.e. to be a Sufficient Function).

### Visual metacognition

Visual metacognition is the ability to evaluate the quality and accuracy of one’s own visual perceptions (Rahnev 2021). Visual metacognition (VM) is what allows us to decide how confident we should be when perceiving things under difficult conditions, such as when we think we might have seen a person moving at the other end of a poorly lit street. Since the representations that VM produces are of visual states, VM is a prima facie candidate for producing our visual experience. Indeed, some researchers have hypothesized that this is a role that it plays (Lau 2019, Fleming 2020).

However, it is unlikely that VM routinely represents as many or the same type of representations as are found in visual experience: as yet, our knowledge of the bandwidth of VM is limited. Experiments investigating VM typically just ask subjects to make judgments concerning no more than one or two perceptions at a time (e.g. Rounis et al. 2010, Fleming et al. 2012, 2014), since the goal of such experiments is not to discover VM’s bandwidth, but rather to discover the conditions under which metacognitive judgments are better or worse (the experimenters often seek correlations between the accuracy of perceptual judgments and subjects’ confidence in them). Nonetheless, it is hard to think of reasons why visual metacognition would produce large numbers of visual representations: the process of assessing one’s own perceptions is a complex task, one that is cognitively and metabolically demanding, and so it would be unexpected if PFC were profligate enough to metacognitively represent each of the many representations found within visual experience. This belies the idea that VM might satisfy the PFC Predictions of Numerosity or Central Vision.

Consider now that, in the case of much of our conscious visual content, it is hard to picture why VM would bother metacognitively assessing them. For example, it is hard to picture why VM would be routinely gauging how confident one should be in, say, one’s perception of a certain level of brightness in the upper-right corner of their visual field. Even less plausible is the idea that VM produces enough metacognitive representations to satisfy the thesis of Expansive Color, since this would require VM to be—at any given moment—metacognitively assessing each of the many hue and/or brightness representations that fill out our visual field. Note that the objection made here is parallel to certain objections against higher-order theories of consciousness, ones saying that it is hard to understand why natural selection would choose to have the human cognitive system re-represent so many mental states (Carruthers 2000, Chaps. 9 & 11).

Overall, VM seems to be a poor candidate for producing enough and the right kind of visual content to fulfill all of the PFC Predictions.

### Even if a PFC function produces enough content, it probably operates in a Lower-deck fashion

As noted earlier, this article’s direct concern is not with PFC Theory in general, but specifically the Upper-deck version, the version according to which it is only when content is actually represented in the PFC that it becomes conscious. This means that, for a PFC function like VWM to help establish that (Upper-deck) PFC Theory is true, whatever content it processes must actually be represented in the PFC; that is, it would not help PFC Theory if the capacity of e.g. working memory was large enough to supply our visual experience, but the representations that it used were all in visual areas, rather than constituted by PFC activity. However, in the case of each PFC function that we’ve been considering, there is reason to think that they operate in a lower-deck fashion.

In the case of VWM, we know that it is probably lower-deck because each of the two most popular theories of VWM hypothesize it to be lower-deck: the debate about the neural basis of VWM is mainly between theories stating that the representations manipulated by VWM are stored exclusively in the visual cortex (Scimeca et al. 2018; Gayet et al. 2018) and those stating that some of them are in the posterior parietal cortex (Xu 2017, 2018); indeed, the idea that all of the representations of VWM are in the PFC is a position that seems to not yet be occupied (see also Block 2007, 2014). And in the case of ensemble representations, i.e. those representations that Ensemble Theory takes to be partially constitutive of visual experience, the relevant evidence suggests that they are constituted in a lower-deck fashion, since their production is associated with activity in inferior temporal and occipital regions (Cant and Goodale 2007; Cant and Xu 2012). Now would be a good time to discuss a recently discovered form of memory, “fragile short-term memory” (Pinto et al. 2013), which has a capacity higher than that of working memory, but which is more susceptible to interference. While its larger capacity might make it a better candidate for producing our visual experience, fragile short-term memory still can’t help PFC Theory here, since fragile short-term memory seems to operate in a lower-deck fashion: among the data supporting this would be neuroimaging evidence indicating its representations to subsist in mid-level visual areas (such as V4) (Sligte et al. 2009), and the fact that transcranial magnetic stimulation to the dorsolateral PFC (an area a known role in working memory) (Curtis and D’Esposito 2003) does not seem to affect the capacity of fragile short-term memory (Sligte et al. 2011).

In the case of VA, its operating in a lower-deck fashion can be gleaned from how one of the main mechanisms by which VA works is through the amplification of activity in visual areas (Moore and Zirnsak 2017), since this suggests that the PFC manipulates working memory representations that are located elsewhere rather than constructing its own. In the case of VM, we do not currently have data concerning whether the visual content that it metacognitively targets is in the PFC or sensory areas. At the same time, both of the leading metacognitive theories of consciousness (Lau 2019, Fleming 2020) hypothesize the type of metacognition that gives rise to visual consciousness to be lower-deck.

Consider now that there is general theoretical reason to doubt that we’ll ever find a PFC function that is not only fecund enough to create all of the representations needed to satisfy the PFC Predictions, but which also operates in an upper-deck manner: there is a classic argument in vision science against theories of vision hypothesizing that the visual system builds up a detailed model of one’s visual environment (Stroud 1956, Minsky 1988; O’Regan 1992, O’Regan and Nöe 2001), one saying that it is unlikely that the visual system would go through the computationally taxing process of building and storing such models, given that all of the information in the visual field is available more or less instantly: to retrieve any of it, one need only make a quick eye movement. The same consideration applies to the PFC functions: the more visual representations that a PFC function uses while performing its job, the more gains in efficiency it gets if the PFC foregoes duplicating the representations to be handled, instead just manipulating those copies of them that already exist in visual areas, thereby letting the visual areas be their “own best model” (cf. Brooks 1991). Overall, it seems unlikely that, even if we found a PFC function that produced the numerous representations required for visual experience, this function would be upper-deck.

In this section, we’ve investigated the issue of whether the PFC might produce as many visual representations as appears in visual experience, doing so by seeing whether there is a PFC function such that it (i) produces all of the representations that would be involved in this and (ii) does so in an upper-deck fashion. It appears that there is no such function (i.e. there is no Sufficient Function).

Of course, it is possible that there is some function of the PFC—as yet undiscovered—that produces those representations appearing in visual experience. This, however, seems unlikely. Given the kind of executive role that the PFC plays in the brain’s cognitive architecture, it would be unexpected if it produced as many representations as is required for filling out our visual experience: The PFC being able to effectively play an executive role in cognition means that it probably must ignore a vast majority of the available visual stimuli so as to focus on just those few that are behaviorally important (Barbas 2009). Indeed, this general idea was confirmed above, when examining those PFC functions that are candidates for producing our visual experience, since each of them seems to be relatively selective as to what they process. As well, for reasons stated just above, it is likely that any PFC function that produces large enough amounts of visual content to satisfy the PFC Predictions would, for sake of efficiency, operate in a lower-deck fashion. Overall, it seems unlikely that there is some PFC function that we have not yet discovered but which is the source of our conscious visual content.

## Conclusions

In this article, we’ve evaluated the idea that the PFC might be an NCC, more precisely, whether it might be the visual content NCC, those brain areas that directly determine ‘what’ we visually experience. What we found is that PFC Theory is hard to reconcile with how visual experience seems in introspection. More specifically, the idea that the PFC is the visual content NCC is hard to reconcile with three theses concerning the nature of visual experience: Numerosity (the typical visual experience contains numerous representations of many types), Central Vision (central vision is especially rich and fine-grained), and Expansive Color (we consciously experience color throughout the visual field). Overall, it seems that, if we look at the functions that the PFC carries out (working memory, attention, etc.), they each don’t produce enough or the right kind of representations for supplying our visual experience, in which case we have tentative reason for thinking that the PFC isn’t the visual content NCC. And, as discussed in the beginning of the article, this has a further consequence: since many forms of HOT Theory seem to entail Upper-deck PFC Theory, the investigation carried out above also gives tentative reason to think that these forms of HOT Theory are false.

I say “tentative” because the argument given above left open the possibility that the anti-richness data (inattentional/change blindness, generic phenomenology, etc.) could yet be enlisted to show that visual experience contains much less content than it seems: due to space constraints, I above only argued for the idea that the Introspective Picture is consistent with anti-richness data—but this leaves open the possibility that, were the data given more scrutiny and analysis, we would find that the best explanation of them is one in which visual experience contains less content than it seems to in introspection. In this way, perhaps PFC Theory could close the gap between how much information visual experience contains and how much visual content the PFC processes. However, given how vast the gulf seems to be between the two, this appears unlikely.

## Data Availability

This article involved the generation of no new data.
