# Correction to: An embarrassment of richnesses: the PFC isn’t the content NCC

**DOI:** 10.1093/nc/niae030

**Published:** 2024-06-27

This is a correction to: Benjamin Kozuch, An embarrassment of richnesses: the PFC isn’t the content NCC, *Neuroscience of Consciousness*, Volume 2024, Issue 1, 2024, niae017, https://doi.org/10.1093/nc/niae017

The following changes have been made to the originally published manuscript.In section **Introduction**, third paragraph, second sentence, text in parentheses should read: “[…][it is a content neural correlate of consciousness (content NCC) (Chalmers 2000)][…]” instead of: “[…][it is a content NCC (Chalmers 2000)],[…]”. And later in the same sentence should read: “[…]it is just that such content can’t become conscious[…]” instead of: “[…]which is just that such content can’t become conscious[…]”.

In the fourth paragraph, end of the last sentence should read: “[…]in which case the PFC can’t be considered the content NCC for vision.” instead of: “[…]in which case the PFC can’t be considered the content neural correlate of visual consciousness (NCC) for vision.”

The emendations have been made in the article.

